# Influence of Membrane Ion Sorption on Ammonium Transport in Donnan Dialysis with Cation Exchange Membranes

**DOI:** 10.1021/acsestengg.5c01024

**Published:** 2026-01-30

**Authors:** Hanyu Tang, Kai Yang, Mohan Qin

**Affiliations:** Department of Civil and Environmental Engineering, 5228 University of Wisconsin-Madison, Madison, Wisconsin 53706, United States

**Keywords:** ammonium recovery, cation exchange membrane, ion sorption, Donnan dialysis

## Abstract

Donnan dialysis (DD) is a promising approach for selectively recovering ammonium ions from wastewater, owing to its simplicity and low energy consumption. However, the role of ion sorption and desorption in cation exchange membranes (CEMs), particularly interactions between ammonium ions (NH_4_^+^) and competing ions (e.g., sodium Na^+^), has often been overlooked. Our experimental results revealed a shift in the Donnan equilibrium caused by the preoccupied counterions in the CEM. For example, when the feed and draw solutions were in a 1:1 concentration ratio, the expected ammonium recovery efficiency was 50%. However, the NH_4_Cl-presoaked membrane resulted in an increase of 19.1 ± 0.5% in the solution NH_4_^+^ concentration and a decrease of 18.8 ± 0.6% in the Na^+^ concentration. Conversely, the NaCl-soaked membrane showed an 18.9 ± 1.6% reduction in NH_4_^+^ and a 23.0 ± 1.3% increase in Na^+^. The difference indicated that the ion exchange capacity of the membrane and counterion uptake could shift the equilibrium of the DD process. We further analyzed the process kinetics and developed a nonsteady-state model incorporating ion sorption capacity to describe the behavior. Our results confirmed that presoaked ions shifted the final DD equilibrium, potentially due to differences in their affinity and geometry. To summarize, this study provides new insights into the mechanisms of Donnan dialysis by accounting for ion sorption and offers insights for the design of more efficient and effective separation processes for ammonium recovery.

## Graphical abstract

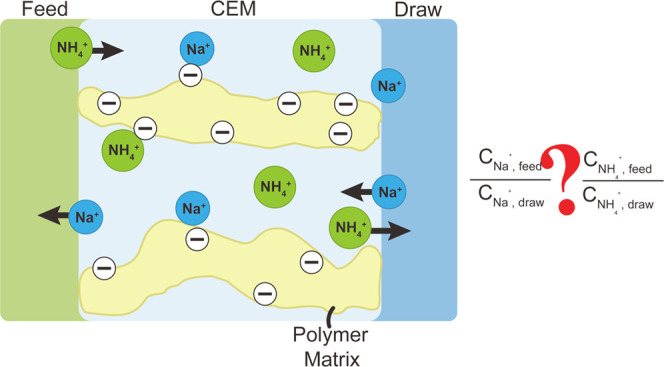

## 1 Introduction

Donnan dialysis has emerged as an effective approach for recovering targeted ions and for removing undesirable ions from aqueous solutions. Distinct from energy-intensive processes relying on additional electrochemical or pressure gradients, Donnan dialysis operates based on a spontaneous electrochemical potential difference between a concentrated driving solution and a feed solution across an ion exchange membrane (IEM).^1,2^ Ions in the feed solution transport across the IEM to the draw solution to maintain the electroneutrality, while the ions in the draw solution move in the opposite direction.^3^ Donnan dialysis gains much attention because of its low energy consumption, though it is a relatively slow process compared to alternative technologies, including membrane stripping, reverse osmosis (RO), and electrodialysis (ED).^4–6^ Since Donnan dialysis intrinsically relies on concentration gradient differences as the driving force, it is particularly suitable as a pretreatment step, enhancing the efficiency of the subsequent energy-intensive process.^7–9^

Donnan dialysis has been successfully employed to remove contaminant ions (e.g., arsenate and fluoride) from water and extract valuable resources such as phosphorus, ammonium, silver, and gold ions.^10–13^ Among these applications, it is a promising technology for the recovery of ammonium (NH_4_^+^) from waste streams. Donnan dialysis has achieved up to 80% ammonium recovery efficiency from synthetic streams with an concentration of 25 mmol L^–1^, proving its potential as a cost-effective ammonium recovery technique.^13^ Furthermore, a hybrid process combining Donnan dialysis with osmotic distillation achieved over 80% recovery efficiency using an alkali solution as the receiver, with low energy consumption of 8.38 and 2.06 kWh kg^–1^ N for initial NH_4_^+^ concentrations of 5 and 50 mmol L^–1^, respectively.^14^ Donnan dialysis coupled with a membrane stripping process successfully recovered 81.4% of NH_4_^+^–N while reducing alkali and acid solution usage by 32.7% and 34.9%, respectively, compared with a conventional membrane stripping process.^5^

Cation exchange membranes (CEMs) containing sulfonate functional groups are commonly employed to selectively transport ammonium ions while excluding anions.^15–17^ The high density of fixed functional groups within CEMs can potentially lead to ion retention within the membrane. Ammonium ions differ significantly from typical monovalent cations, such as sodium (Na^+^) and potassium (K^+^), due to their more complex tetrahedral geometry. This structure complexity comes from the presence of lone pair electrons, leading to different electron densities that may influence the interaction between ammonium ions and the fixed charge groups and consequently affect their transport behavior within the membrane.^18^ Consequently, this kind of transport property of ammonium ions can lead to unbalanced ion concentrations between the membrane phase and the external solution.

On the other hand, previous studies have successfully predicted the salt permeability coefficients of ion exchange membranes in contact with aqueous solutions using the solution–diffusion framework. Complementary approaches, such as the Manning diffusion model and Mackie–Meares model, were used to consider the ion diffusion within hydrated CEMs.^19,20^ Meanwhile, the kinetics of Donnan dialysis processes have been extensively investigated for applications in nutrient recovery, contaminant removal, and solution purification.^21,22^

Although the ion sorption behavior of CEMs has been widely characterized, discrepancies often remain between experimentally measured equilibrium ion concentrations and predicted ones by ideal Donnan-based descriptions.^23,45^ These deviations arise not because the equilibrium state within the membrane phase does not fully capture the underlying interactions but because commonly used simplified models for ion partitioning oversimplify how different ions share and compete for fixed charges in the membrane. Such equilibrium deviations have received limited attention in previous studies, despite their significance for accurately modeling ion transport within the membrane during the Donnan dialysis process. These deviations highlight the importance of solution membrane interactions and competitive cation partitioning, which is highly relevant to Donnan dialysis applications where the choice of draw solution plays a critical role in recovery performance. Moreover, the existing mathematical theory of Donnan dialysis often combines idealized equilibrium assumptions with further simplification in transport, which can leave systematic differences between predicted and observed ion concentrations in each chamber. Addressing how realistic equilibrium partitioning and membrane sorption feed into transient transport, therefore, remains important for refining models of CEM behavior in Donnan dialysis.

In this study, we investigate how the sorption and desorption behaviors of a cation exchange membrane affect NH_4_^+^ transport, especially when Na^+^ serves as the extracting ion, with the aim of explicitly addressing the mismatch between ideal model predictions and experimental observations. We first compare predictions from an ideal Donnan dialysis description with experimentally normalized results obtained from the dialysis cell to quantify how membrane presoaking and ion exchange capacity shift NH_4_^+^/Na^+^ partitioning between the membrane and the solutions. To further address this mismatch, a nonsteady state model was used to describe the kinetics of the process, including the sorption behavior in the membrane. In addition, we propose a modified Donnan dialysis equation connecting the initial influent concentration to the final equilibrium state, accounting for the impact of ion sorption on the presoaked membrane. Finally, we compared ion concentration ratios in the membrane and in the solutions to gain deeper insight into the possible mechanism responsible for the observed discrepancies, which is relevant for improving the interpretation and modeling of ion transport in membrane processes.

## 2 Materials and Methods

### 2.1 Materials and Chemicals

Cation exchange membrane (CEM) was purchased from Membranes International Inc. (CMI-7000S, NJ, USA) and used for all experiments. The membrane properties are listed in Table 1. CEM membrane was immersed in 1 mol L^–1^ NaCl solution or 1 mol L^–1^ NH_4_Cl solution for 24 h to be saturated with Na^+^ or NH_4_^+^ ions, respectively. Prior to use, the membrane was rinsed three times with deionized water to remove excess ions on the surface.

**Table 1:** Characterization of Cation Exchange Membrane (CMI-7000)^a^

parameter	CMI-7000
ion exchange capacity, meq g^–1^ dry membrane^a^	1.6 ± 0.1
water uptake, g water g^–1^ dry membrane	0.261 ± 0.04
thickness, μm	450 ± 10
permselectivity, %, 0.1 mol KCl/kg/0.5 mol KCl/kg	94
electric resistance, Ohm cm^2^, 0.5 M NaCl	<30

a From manufacture information.

### 2.2 Donnan Dialysis Reactor

The Donnan dialysis (DD) reactor was constructed from square polycarbonate plates with inner rubber frames, forming separated feed and draw chambers divided by a cation exchange membrane with an effective membrane area of 64 cm^2^, where the ion exchange capacity is 2.00 ± 0.21 mequiv g^–1^, as confirmed by titration experiments in previous study.^18^ During the experimental process, membrane reformation did not appear to significantly affect the solution volume in the feed and draw chambers. The rubber frames were sandwiched between the polycarbonate plates to create the internal chamber, while the membrane was positioned freely between the rubber plates without any backing support in the effective area. The volumes of the feed and draw chambers were both 250 mL. The recirculation rate in each chamber was 115 mL min^–1^. The cycle duration time was 24 h. To investigate the Donnan dialysis recovery efficiency, 50 mM of NH_4_Cl was used to simulate a high ammonium wastewater stream, and 100 mM of NaCl was chosen as the draw solution to provide a constant counterion flow.^1,13^ To further investigate the exchange of NH_4_^+^ with Na^+^, the DD reactor was filled with NH_4_Cl in the feed with varying concentrations from 10 mmol L^–1^ NH_4_^+^ to 200 mmol L^–1^ NH_4_^+^, and NaCl in the draw ranged from 25 mmol L^–1^ to 200 mmol L^–1^. The concentrations of Na^+^ and NH_4_^+^ ions were measured via ion chromatography (IC, Thermo Dionex 2100 Ion Chromatography System, Thermo Fisher Scientific, Waltham, MA, US).

### 2.3 Ion Desorption

To quantify the cation concentrations in CEM, the membrane was first immersed in 1 mol L^–1^ NaCl or 1 mol L^–1^ NH_4_Cl for at least 24 h, followed by immersion in 0.1 mol L^–1^ KNO_3_ to exchange the sodium or ammonium ions retained in the membrane with potassium ions from the solution. Additionally, to measure the ion concentrations in CEM after the Donnan dialysis process, the residual solution on the membrane surface was carefully removed. Then the membrane was soaked in 50 mL of 0.1 mol L^–1^ KNO_3_ to exchange the sorbed ions for at least 24 h. This concentration was chosen to provide a several-fold excess relative to the fixed charge group content. To promote effective desorption of exchangeable cations, the desorption process was repeated twice.^24,25^ Although absolute complete exchange cannot be strictly guaranteed, this approach provides a consistent and reproducible basis for comparing relative amounts of sorbed cations between different presoaked solutions after the Donnan dialysis process using the same feed and draw solution. The ion concentration in the membrane is defined as the moles of ions per L of membrane-sorbed water.

### 2.4 Water Uptake

Water uptake of the CEM membrane was measured gravimetrically at room temperature.^26^ First, the membrane was immersed in deionized water for at least 24 h to ensure thorough hydration. After soaking, the membrane was removed from the water, and any excess surface water was carefully wiped using Kimwipe. After measuring the wet weight of the membrane (*m*_wet_), the membrane was then dried under vacuum at room temperature for 48 h to achieve a stable dry weight (*m*_dry_). The water uptake (*W*_u_) was calculated using the following equation^27^

1 $$W_{u}=\frac{m_{wet}-m_{dry}}{m_{dry}}$$

## 3 Model Development

### 3.1 Modified Donnan Dialysis Model

The model developed in this study is based on a 24 h batch experiment conducted in a Donnan dialysis cell consisting of two chambers: the feed (*f*) and draw (*d*) compartments. In the model, co-ions are assumed to be completely excluded from the membrane, and the water content is considered constant throughout the experiment. The self-diffusion coefficient of ions within the membrane inherently accounts for all membrane-related factors and specific ion–polymer interactions. The effect of osmotic pressure gradients and the convective osmotic water flow are neglected due to their relatively minor contribution under the experimental conditions. Both the feed and draw compartments were continuously recirculated to minimize the thin film layer at the membrane surface. Under these hydrodynamic conditions, we treat the bulk concentrations as uniform and assume that membrane diffusion dominated the ion exchange process, although some residual boundary layer resistance may remain. The detailed derivation is provided in Text S1, and the following section highlights the main steps.

At Donnan equilibrium, the electrochemical potential (μ̅) of a typical charged species, *i*, in the feed (*f*) and draw (*d*) side at time *t*, is equal:

$$\overset{-}{\mu_{i}^{f}}\,=\overset{-}{\mu_{i}^{d}}$$ 2

This can be expressed as

3 $$\frac{z_{i}F\left(\psi_{d,t}-\psi_{f,t}\right)}{RT}=\ln\left(\frac{a_{i,f,t}}{a_{i,d,t}}\right)$$

where *R* is the gas constant, *a* is ionic activity, *z* is the ion valence, *F* is Faraday’s constant, ψ is the electrical potential, and *i* can be Na^+^ or NH_4_^+^. We approximate activities by concentrations and do not explicitly account for the activity coefficient. Under this simplification, eq 4 describes the relationship between feed and draw concentrations for Na^+^ and NH_4_^+^ at Donnan equilibrium:

4 $$\frac{C_{Na_{f,t}^{+}}}{C_{Na_{d,t}^{+}}}=\frac{C_{N{H_{4}^{+}}_{f,t}}}{C_{N{H_{4}^{+}}_{d,t}}}$$

Because the volume ratio of the solution in the feed side to draw side is equal, the equation at Donnan equilibrium can be written as

5 $$\frac{n_{Na_{f,t}^{+}}}{n_{Na_{d,t}^{+}}}=\frac{n_{N{H_{4}^{+}}_{f,t}}}{n_{N{H_{4}^{+}}_{d,t}}}$$

At the Donnan equilibrium, the same deduction can be applied to any pair of phases (feed, draw, or membrane), for which the partitioning factor is identical for Na^+^ and NH_4_^+^. Consequently, the concentration ratio is conserved among the feed, draw, and membrane phases, leading to the following relationship:

6 $$\frac{n_{Na_{m,t}^{+}}}{n_{Na_{f,t}^{+}}}=\frac{n_{N{H_{4}^{+}}_{m,t}}}{n_{N{H_{4}^{+}}_{d,t}}}$$

when the membrane is presoaked in NaCl solution, we assume that only Na^+^ is present in the membrane. Here, Δ*n* *i_t_* denotes the net change of specie *i* due to ion exchange with the membrane up to Donnan equilibrium. A positive Δ*n* *i_t_* indicates a net release from the membrane to the solution, whereas a negative value indicates a net uptake by the membrane

7 $$\Delta n_{Na_{t}^{+}}=IEC-n_{Na_{m,t}^{+}}$$

8 $$\Delta n_{N{H_{4}^{+}}_{t}}=-n_{N{H_{4}^{+}}_{m,t}}$$

These equations follow directly from ion conservation in the membrane, where the total amount of fixed charge groups is constant. Accordingly, NH_4_^+^ uptake by the membrane corresponds to an equal loss from the solution phase. Under an ideal Donnan equilibrium, Na^+^ and NH_4_^+^ have the same Donnan potential. As a result, their concentration ratios are identical across the membrane solution phases. Consequently, the molar ratio of Na^+^ to NH_4_^+^ remains the same inside the membrane and the solution. Based on the relationship, eq 6 can be further written as the following

9 $$\frac{n_{N{H_{4}^{+}}_{m,t}}}{n_{Na_{m,t}^{+}}}=\frac{n_{N{H_{4}^{+}}_{total,t}}}{n_{Na_{total,t}^{+}}}=\frac{n_{N{H_{4}^{+}}_{f,0}}+\Delta n_{N{H_{4}^{+}}_{t}}}{n_{Na_{d,0}^{+}}+\Delta n_{Na_{t}^{+}}}=\frac{n_{N{H_{4}^{+}}_{f,0}}+\Delta n_{N{H_{4}^{+}}_{t}}+n_{N{H_{4}^{+}}_{m,t}}}{n_{Na_{d,0}^{+}}+\Delta n_{Na_{t}^{+}}+n_{Na_{m,t}^{+}}}$$

The equation can be further modified according to eqs 7 and 8, the final ratio of NH_4_^+^ to Na^+^ in the solution can be predicted using

10 $$\frac{n_{N{H_{4}^{+}}_{total,t}}}{n_{Na_{total,t}^{+}}}=\frac{n_{N{H_{4}^{+}}_{f,0}}}{n_{Na_{d,0}^{+}}+IEC}$$

when the membrane is presoaked by NH_4_Cl, the final ratio of NH_4_^+^ to Na^+^ in the solution can be written as, the detailed process of deduction is shown in Text S1

11 $$\frac{n_{N{H_{4}^{+}}_{total,t}}}{n_{Na_{total,t}^{+}}}=\frac{n_{N{H_{4}^{+}}_{f,0}}+IEC}{n_{Na_{d,0}^{+}}}$$

### 3.2 Ammonium Transport Kinetics

In many Donnan dialysis analyses, one ion is assumed to be in large excess, and the system is reduced to Fick’s first law under a steady-state approximation.^28,29^ In this work, we instead adopt a modeling framework that integrates the Nernst–Planck description with with the transient form of Fick’s second law, without imposing dilution in the assumption. The kinetics of ion transport in Donnan dialysis were modeled to obtain ion concentration profiles inside the membrane as functions of both time and membrane thickness. The detailed derivation of the process has been reported previously.^30^ This model, uniquely tailored to capture the dynamics of specific ions, such as NH_4_^+^ and Na^+^, is applied to predict their transport across the membrane in the DD process.When CEM was presoaked in 1 mol L^–1^ NH_4_Cl, Na^+^ concentration in the membrane can be written as:^30^

12 $$\frac{\partial y_{{Na}^{+}}\left(\epsilon ,\tau \right)}{\partial \tau }=\frac{\partial }{\partial \epsilon }\left[D\left(y_{{Na}^{+}}\right)\frac{\partial y_{{Na}^{+}}\left(\epsilon ,\tau \right)}{\partial \epsilon }\right]$$

where *y*_Na_^+^(ϵ,τ) represents the mole fraction of Na^+^ at time (τ) and x-coordinate (ϵ) normalized by CEM ion exchange capacity. For brevity, the dependence on (ϵ,τ) is omitted. *D*(*y*_Na_^+^) is the interdiffusion coefficient at position ϵ inside the membrane and time τ in terms of dimensionless quantities. Based on the definition of the interdiffusion coefficient, which quantifies the diffusion rate of specific species in a mixed solution, *D*(*y*_Na_^+^) can be written as,^3,31^

$$D\left(y_{{Na}^{+}}\right)=\frac{1}{1+\left(\frac{\overset{®}{D_{{Na}^{+}}\;}}{\overset{®}{D_{{NH}_{4}^{+}}\;}}-1\right)y_{{Na}^{+}}}$$ 13

where $\overset{®}{D_{i}\;}$ is the diffusion coefficient of specie *i* in the membrane.

The boundary condition is determined from the mass balance requirement: the rate of ions leaving the membrane is equal to the rate of ions entering the membrane. Therefore, the concentration inside the membrane can be written as follows:

14 $$\frac{V}{S}\frac{\partial C_{{Na}^{+}}}{\partial t}=\overset{®}{D_{{Na}^{+}{NH}_{4}^{+}}\;}\frac{\partial \overset{®}{C_{{Na}^{+}}\;}}{\partial x}$$

where *C*_*i*_ is the concentration of ion *i* in solution (feed + draw), *S* is the effective membrane surface area, $\overset{®}{D_{{Na}^{+}{NH}_{4}^{+}}\;}$ is the interdiffusion coefficient in the membrane, and $\overset{®}{C_{i}\;}$ is the concentration of specie *i* in the membrane.

To quantitatively describe the membrane solution partitioning of Na^+^ and NH_4_^+^, a separation factor is introduced and defined as the ratio of the Na^+^/NH_4_^+^ concentration ratio in the membrane to that in the bulk solution

15 $$K_{{NH}_{4}^{+}}^{{Na}^{+}}=\frac{\overset{®}{C_{{Na}^{+}}\;}}{C_{{Na}^{+}}}\frac{C_{{NH}_{4}^{+}}}{\overset{®}{C_{{NH}_{4}^{+}}\;}}$$

Using eqs 13 and 14, the solution concentration can be determined from the separation factor and the constant total salt concentration in each chamber. Consequently, eq 14 can thus be further modified as

16 $$\frac{\partial y_{{Na}^{+}}}{\partial \tau }=\left(\frac{Q}{VC_{T}}\right)\frac{(K_{{NH}_{4}^{+}}^{{Na}^{+}}-\left(K_{{NH}_{4}^{+}}^{{Na}^{+}}-1\right)y_{{Na}^{+}}{C_{{Na}^{+}})}^{2}}{K_{{NH}_{4}^{+}}^{{Na}^{+}}}\left(\frac{1}{1+\left(\frac{D_{{Na}^{+}}}{D_{{NH}_{4}^{+}}}-1\right)y_{{Na}^{+}}}\right)\frac{\partial y_{{Na}^{+}}}{\partial \epsilon }$$

where *Q* is the ion exchange capacity of the effective membrane area, $K_{{NH}_{4}^{+}}^{{Na}^{+}}$ is the separation factor, and *C*_*T*_ is the total cation concentration in the system (*C*_*f*_ + *C*_*d*_). As a result, the final cation concentration in the feed chamber can be written as

17 $$C_{{Na}^{+},f}\left(\tau +\Delta \tau \right)=C_{T,f}\frac{c_{{Na}^{+}}\left(0,\tau +\Delta \tau \right)}{K_{{NH}_{4}^{+}}^{{Na}^{+}}-c_{{Na}^{+}}\left(0,\tau +\Delta \tau \right)\left(K_{{NH}_{4}^{+}}^{{Na}^{+}}-1\right)}$$

The ammonium concentration can be calculated through the unchanged anion concentration, which can be written as

18 $$C_{{NH}_{4}^{+},f}\left(\tau +\Delta \tau \right)=C_{T,f}-C_{{Na}^{+},f}\left(\tau +\Delta \tau \right)$$

when the membrane was presoaked by NaCl, it can be written as

19 $$C_{{NH}_{4}^{+},f}\left(\tau +\Delta \tau \right)=C_{T,f}\frac{c_{{NH}_{4}^{+}}\left(0,\tau +\Delta \tau \right)}{K_{{Na}^{+}}^{{NH}_{4}^{+}}-c_{{NH}_{4}^{+}}\left(0,\tau +\Delta \tau \right)\left(K_{{Na}^{+}}^{{NH}_{4}^{+}}-1\right)}$$

20 $$C_{{Na}^{+},f}\left(\tau +\Delta \tau \right)=C_{T,f}-C_{{NH}_{4}^{+},f}\left(\tau +\Delta \tau \right)$$

## 4 Results and Discussion

### 4.1 Characteristics of Cation Exchange Membrane

The cation exchange membrane (CMI-7000) we used throughout this study is a dense nonporous polymer film. Table 1 summarizes the main properties of CMI-7000. The membrane contains sulfonate groups as functional groups and has a nominal ion exchange capacity of 1.6 ± 0.1 mequiv g^–1^ dry membrane. Salt sorption tests were conducted with both Na^+^ and NH_4_^+^ solutions. When the membrane was presoaked in 1 mol L^–1^ NaCl solution for at least 24 h, the sodium ion concentration inside the membrane reached 1.78 ± 0.05 mmol g^–1^ dry membrane (used throughout the analysis). In contrast, being immersed in 1 mol L^–1^ of NH_4_Cl solution resulted in a slightly smaller sorption capacity of 1.62 ± 0.06 mmol g^–1^ dry membrane. The results demonstrate that counterion uptake in the membrane depends on the type of external salt, even when the salt concentrations are the same.^32^

The slight difference in ion uptake capacity between Na^+^ and NH_4_^+^ concentrations might be related to the hydration abilities of ions and their affinities to the sulfonate functional group in CEM. As shown in Table 2, the hydration energy and hydration radius of Na^+^ are 87.2 kcal mol^–1^ and 3.58 Å, respectively, and both are higher than them for NH_4_^+^. NH_4_^+^ has a higher adsorption preference than sodium on CEMs, which can be attributed to its smaller hydration radius and lower hydration energy.^18^ However, Na^+^ concentrations in the CEM exceeded those of NH_4_^+^, even though the smaller solvated equivalent ions were more favorable. NH_4_^+^ has a tetrahedral geometry and an uneven distribution of electron density, resulting in a possible steric hindrance in the channel of the membrane and a reduced amount of adsorbed NH_4_^+^ in the membrane compared with Na^+^.^18,33^ These findings suggest that the internal ion concentration within the membrane can impact the elution behavior of cations in the Donnan dialysis process with potential implications for selective ion recovery (Figure 1a).

**Table 2:** Ionic Radius, Hydration Radius, Hydration Energy, and Hydration Number of Sodium and Ammonium

parameters	Na^+^	NH_4_^+^
ionic radius, Å	1.02	1.48
hydration radius, Å^34^	3.58	3.31
hydration energy, kcal mol^–1^ ^35^	87.2	68.1
hydration number	1.5	0.4

**Figure 1:**
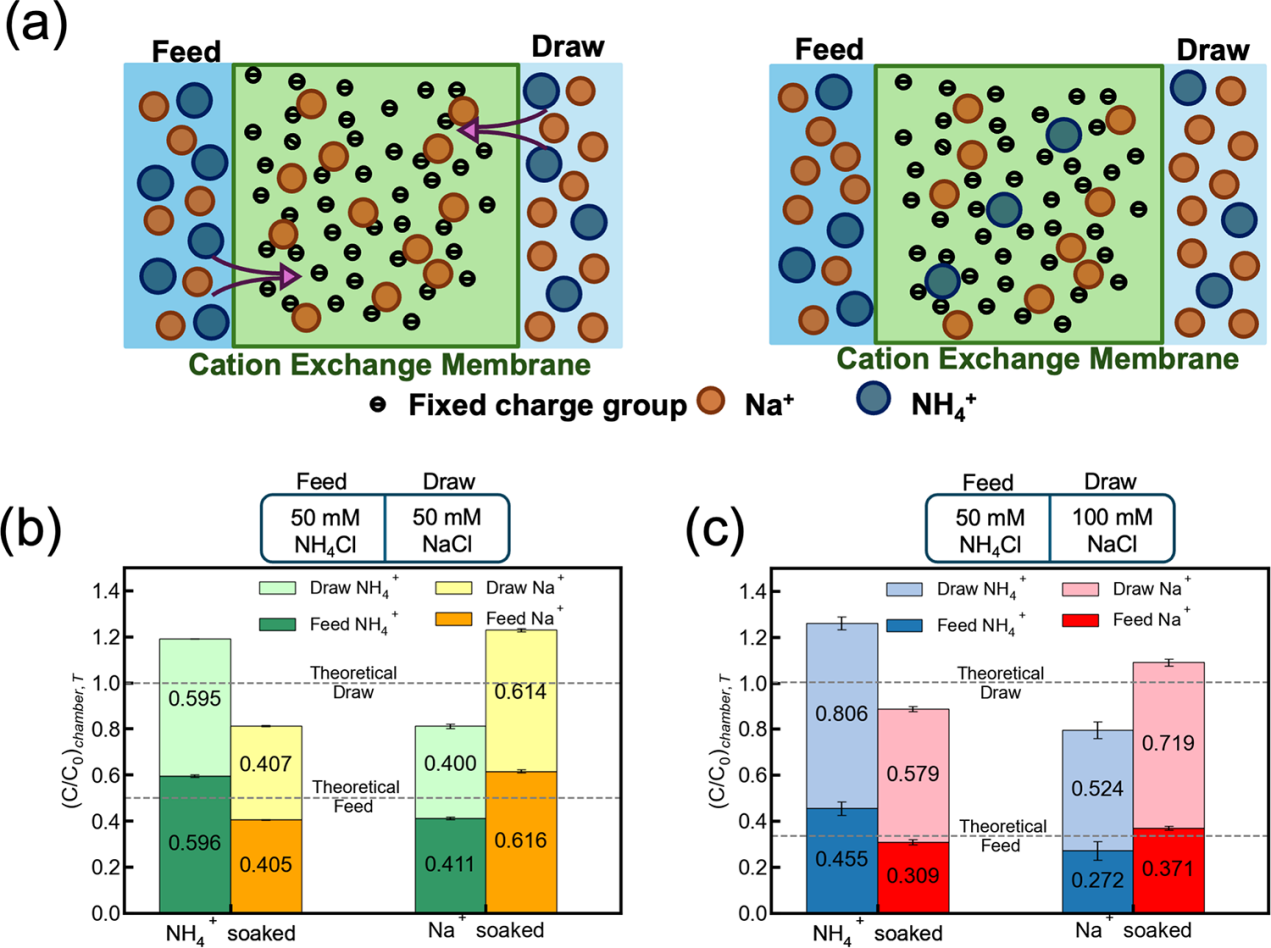
(a) The schematic of Donnan dialysis with the membrane presoaked in Na^+^ ions. (b) The normalized concentration ratios of NH_4_^+^ and Na^+^ in different chambers (feed or draw) when *t* = T is 24 h, and the volume of each chamber was 250 mL. The dash line represents the theoretical number when using ideal Donnan dialysis (eq 4) to calculate the proportion of each ion in each chamber. The CEMs were soaked in 1 mol L^–1^ NH_4_Cl or NaCl solution for 24 h prior to the experiment. (b) The initial feed solution was 50 mmol L^–1^ NH_4_Cl, and the draw solution was 50 mmol L^–1^ NaCl. (c) The initial feed solution was 50 mmol L^–1^ NH_4_Cl, and the initial draw solution was 100 mmol L^–1^ NaCl.

### 4.2 Unbalanced Ion Distributions at Equilibrium

To evaluate the impact of presoaked ions within the membrane on the cation distribution in both chambers, DD experiments were conducted using Na^+^ and NH_4_^+^ solutions, and the concentrations of all cations were analyzed (Figure 1). Two conditions were tested: one experiment has 50 mmol L^–1^ NH_4_Cl and 50 mmol L^–1^ NaCl in the feed and draw chambers, respectively (Figures 1b and S1), while the other has a feed solution of 50 mmol L^–1^ NH_4_Cl and a draw solution of 100 mmol L^–1^ NaCl (Figures 1c and S2). Prior to the experiments, the membrane was presoaked in either 1 mol L^–1^ NH_4_Cl or NaCl solution for at least 24 h.

Figure 1b presents the discrepancy between the theoretical and practical ion distributions in each chamber at equilibrium when the feed and draw chambers contain 50 mmol L^–1^ NH_4_Cl and NaCl, respectively. Theoretically, if the membrane ion uptake is neglected in the DD process, then the normalized concentrations of both Na^+^ and NH_4_^+^ are 25 mmol L^–1^ in both feed and draw chambers. This theoretical case therefore serves only as a simple ideal reference that neglects membrane sorption. In our experiments, the NH_4_Cl-presoaked membrane resulted in an increased solution NH_4_^+^ amount by 19.1 ± 0.5% and a decreased Na^+^ amount by 18.8 ± 0.6%. In contrast, the NaCl-soaked membrane showed an 18.9 ± 1.6% reduction in the level of NH_4_^+^ and a 23.0 ± 1.3% increase in the level of Na^+^. The difference indicates that membrane sorption capacity and counterion uptake could shift the equilibrium of the DD process.

We observed the same trend when initial concentrations in the feed and draw were set at 50 and 100 mmol L^–1^, respectively (Figure 1c). When the membrane was pretreated with 1 mol L^–1^ of NH_4_Cl, the total amount of NH_4_^+^ increased by 26.1 ± 5.8%, indicating that NH_4_^+^ ions were released from the membrane to solutions. Simultaneously, the total amount of Na^+^ decreased by 11.2 ± 2.4% due to Na^+^ being adsorbed by the membrane. Conversely, with a NaCl-soaked membrane, the NH_4_^+^ concentration decreased by 20.4 ± 7.6% and the Na^+^ concentration increased by 9.0 ± 2.5%. These results suggest that the presoaked CEM function as ion reservoirs, with cations in the membrane actively participating in the DD process. Furthermore, the cation exchange between the membrane and solutions affects the final distribution of the cations in both chambers. The total cation concentration whichever membrane was presoaked increased, while it decreased in the other. The overall Donnan dialysis equivalent in the system changes based on all the free cations in the solution.

Figure 1b,c also highlights the deviation in the proportion between Na^+^ and NH_4_^+^ at equilibrium. Theoretically, when both the feed and draw contain 50 mmol L^–1^ concentrations of NH_4_Cl and NaCl, respectively, the equilibrium proportion of each cation in both feed and draw chambers is expected to be 0.5 (i.e., 25 mmol L^–1^ Na^+^ and 25 mmol L^–1^ NH_4_^+^). However, the experimental results show that the percentage of NH_4_^+^ in the NH_4_Cl-presoaked membrane is higher than 0.500, which is 0.596 and 0.595 in the feed and draw chambers, respectively. A similar trend is also shown when the feed and draw contain 50 mmol L^–1^ NH_4_Cl and 100 mmol L^–1^ NaCl. The experimental percentage of NH_4_^+^ in the NH_4_Cl-presoaked membrane is higher than the theoretical numbers 0.333 and 0.667, which are 0.455 and 0.806 in the feed and draw chambers, respectively. In contrast, the Na^+^ percentage is lower than expected (i.e., 0.379 in feed and 0.579 in draw solution). The observed changes in the two cation concentrations after the experiment, both increases and decreases, indicate the ratio between the solutions.

The ratio of each cation between different chambers remained constant, regardless of the presoaked solution. The experimental results show that the ratio of NH_4_^+^ between feed and draw chambers was 1.001 ± 0.007 and 1.026 ± 0.029 in NH_4_^+^- and Na^+^-presoaked solution, respectively. A similar trend was observed when the feed contains 50 mmol L^–1^ NH_4_Cl and the draw with 100 mmol L^–1^ NaCl. The equilibrium ratio of NH_4_^+^ in feed to draw chamber is 0.564 ± 0.019 and 0.518 ± 0.054, when presoaked by NH_4_^+^ and Na^+^, respectively, when the ideal number is 0.5. This phenomenon indicates that even though the total amount of the cations changed, the ratio of each part in the solution remained the same under equilibrium, which is held by the electrochemical gradient difference (eq 3).

These large deviations between the experimental number and the theoretical results from neglecting ion adsorption and exchange in CEM (Figure S3). Although predictions based on initial concentrations provide a rough estimate, they require adjustments to account for membrane interactions. The IEM therefore acts as an additional reservoir that modifies the effective equilibrium state of the system, highlighting the need for a more comprehensive description that includes both membrane sorption and transport dynamics.

### 4.3 The Kinetics of Donnan Dialysis Process in Solution and Membrane

To further investigate how ion uptake in the membrane impacts the Donnan dialysis process, we measured ion concentrations as a function of time in both feed and draw chambers with a NH_4_^+^: Na^+^ratio of 1:2. Initially, the feed solution contained 50 mmol L^–1^ of NH_4_Cl, and the draw solution had 100 mmol L^–1^ of NaCl. The membranes were presoaked in either 1 mol L^–1^ NaCl or 1 mol L^–1^ NH_4_Cl solutions. Each experiment ran for 24 h, as equilibrium was reached within that duration. The solution concentration in Figure 2a,b reflects the combined concentration of a specific cation in both feed and draw chambers (calculated as eq S18). We used concentration as the unit because the concentration gradient across the membrane is the driving force for ion transport. Since both chambers have equal volumes (250 mL each side), the concentration directly reflects the total amount of ions.

**Figure 2:**
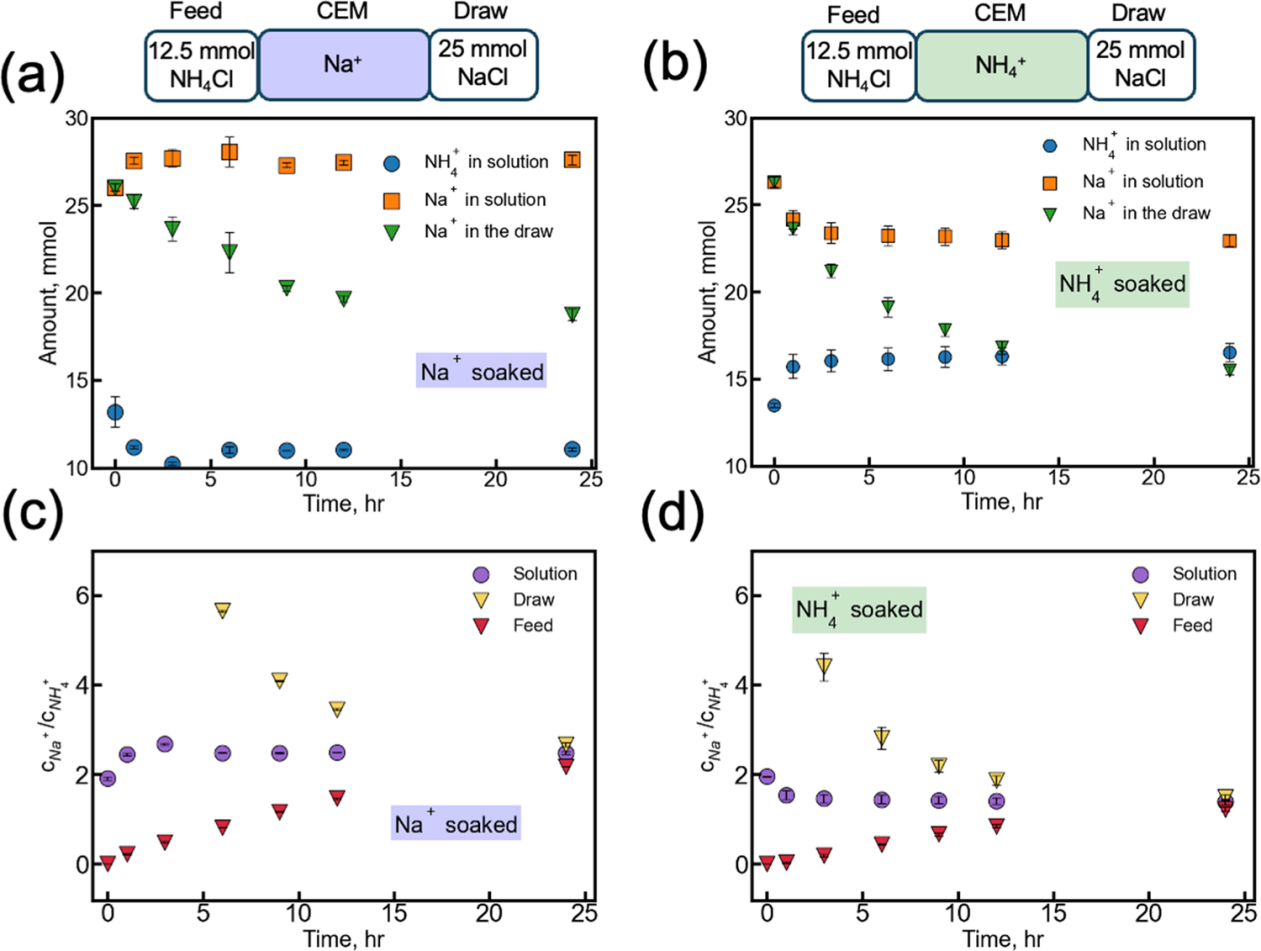
Amounts and the ratios of Na^+^ to NH_4_^+^ profiles in Donnan dialysis processes as a function of time. The feed solution is 50 mmol L^–1^ NH_4_Cl, while the draw solution is 100 mmol L^–1^ NaCl. (a) and (b) show the total amount of NH_4_^+^ (blue dots) and Na^+^ (orange square) in the solution (feed + draw), along with the amount of Na^+^ in the draw side (green triangle), as a function of time when the membranes were presoaked in 1 mol L^–1^ NaCl (a) and 1 mol L^–1^ NH_4_Cl (b). (c,d) show the ratio of Na^+^ to NH_4_^+^ in the solution over time, with presoaking in 1 mol L^–1^ NaCl (c) and 1 mol L^–1^ NH_4_Cl (d). In (c,d), purple dots represent the ratio in the total concentration in both chambers (draw + feed), yellow triangles indicate the ratio in the draw solution, and red triangles represent the feed solution.

Figure 2a,b illustrates the total cation concentrations in the solutions as a function of time when the membrane was presoaked in 1 mol L^–1^ of NaCl or NH_4_Cl solutions, respectively. When the membrane was presoaked in 1 mol L^–1^ Na^+^, the total amount of NH_4_^+^ in the solution decreased to 77.5%, while Na^+^ increased by 6.4% during the first 3 h. The same trend was observed when the membrane was presoaked in 1 mol L^–1^ NH_4_^+^: during the first 3 h, Na^+^ decreased to 11.1% when NH_4_^+^ increased by 19.1%. In both scenarios, the total amounts in the solutions stabilized after 3 h, whereas the cation amount in the chamber continued to change, indicating that the overall system equilibrium was not yet reached. This phenomenon suggests that different regions of the system approach equilibrium on different time scales. After the initial stage, the ionic content of the membrane reached a steady state, which did not further uptake or release ions, particularly when the initial ionic compositions differed between the membrane and the solutions. The interdiffusion, where different species (Na^+^ and NH_4_^+^) diffuse mutually across the membrane, affects the cation transport during the first 3 h. Nonsteady-state modeling of the membrane content (Figures S6 and S7) shows that the cation content inside the membrane reached near-steady state within the first 0.5 h. Consequently, the total amount of cation in the solution (feed + draw) stabilizes rapidly. The small further changes observed experimentally between 1 and 3 h arise from the coarse sampling intervals. Subsequent changes were minor compared to the ongoing slower adjustments between two chambers. These observations indicate that different regions of the system equilibrate on different time scales, where the membrane interior equilibrates rapidly, whereas the solution in the chambers continues to adjust over time. Once the membrane reached steady-state, further uptake or release of cations became negligible, and the slower equilibration in the bulk solution determined the later stage of the process.

The total ion concentrations of each ion stabilize in solution after 3 h in both scenarios (Figure 2a,b). This observation suggests that ion uptake by the membrane approaches a steady state earlier than the stabilization of ion concentrations in the bulk solution. This difference in time scales arises because different regions in the system reach steady conditions at different rates. Compared to the bulk solutions, the membrane acts as a reservoir with a much smaller capacity, and its limited thickness results in a shorter time to reach a steady state. Even though the membrane selectivity toward Na^+^ and NH_4_^+^ is different, the localized interdiffusion coefficient primarily depends on the minority species.^3^ In both cases, the minority cation, either Na^+^ or NH_4_^+^, has the same magnitude of the diffusion coefficient in the membrane (0.301 ∼ 0.605 × 10^–9^ m^2^ s^–1^ and 0.103 × 10^–9^ m^2^ s^–1^ for Na^+^ and NH_4_^+^, respectively).^36,37^ Consequently, the equilibration times between the membrane and solution remain comparable in both scenarios. In the absence of the external applied electric field, ion diffusion across the membrane is primarily governed by two factors: membrane diffusion and boundary layer diffusion, with the internal membrane potential implicitly determined by electroneutrality constrains.^3^ Previous studies have quantified these factors using resistance to evaluate the relative contributions of both factors during the dialysis process.^38^ Initially, ion transport is primarily driven by membrane uptake due to the steep concentration gradient between the bulk solution and the membrane phase. This effect is most substantial during the first 3 h, as the membrane uptake increases rapidly. As membrane ion uptake reached a steady state, the contribution of membrane diffusion to the overall flux decreased and interfacial mass transfer resistance associated with the boundary layer became more important.

The ion uptake inside membranes also impacts the concentration ratio of sodium to ammonium in solutions. As shown in Figure 2c,d, the initial Na^+^/NH_4_^+^ ratio in the Na^+^-presoaked membrane was 1.91 ± 0.04, which increased to 2.48 ± 0.03 after 24 h. A similar trend was observed for membrane presoaked by NH_4_^+^, where the Na^+^/NH_4_^+^ ratio decreased from 1.95 ± 0.01 to 1.39 ± 0.06 after 24 h. According to eq 11, the theoretical Na^+^/NH_4_^+^ ratio should be 1.39 ± 0.02 and 1.42 ± 0.01 for Na^+^-presoaked and NH_4_^+^-presoaked membranes, respectively, when membrane sorption was taken into account. The initial Na^+^/NH_4_^+^ ratio deviates from the theoretical value but gradually approaches the predicted ratio at equilibrium, as shown in Figure 2c,d. At equilibrium, the Na^+^/NH_4_^+^ ratios in the feed, draw solution, and membrane converge due to the need to satisfy electrochemical potential equilibrium across all three domains. This behavior indicates that the new equilibrium is established through ion sorption and exchange between the solution and membrane, resulting in the distribution of ions in the solution.

### 4.4 Modified Donnan Dialysis Model

IEM sorption plays a significant role in the Donnan dialysis process, particularly when the membrane retains a quantity of counterions comparable to the amount of ions present in the solutions. Therefore, taking membrane sorption into account for Donnan dialysis is needed. While previous studies have extensively investigated ion transport kinetics within the membrane and solution, few have examined how sorption impacts the final equilibrium throughout the process. Neglecting ion sorption in the membrane can shift equilibrium concentrations and lead to inaccuracies in calculating ion distributions.^30,38,39^ To address this, we modified the conventional DD model to explicitly incorporate membrane sorption, thereby enhancing the accuracy of predicting the final ion distributions. The specific parameters used in the modeling is listed in Table S1.

To validate the modified model, experiments were conducted by using varying ion concentration ratios. For membranes presoaked in 1 mol L^–1^ NH_4_Cl solution, the NH_4_Cl in the feed chamber was fixed at 50 mmol L^–1^, while the NaCl concentration varied from 10 mmol L^–1^ to 200 mmol L^–1^ in the draw solution. Conversely, for membranes presoaked in 1 mol L^–1^ NaCl, the NaCl concentration was fixed at 50 mmol L^–1^ in the feed, and the NH_4_Cl concentration was adjusted from 10 mmol L^–1^ to 200 mmol L^–1^ in the draw solution. The ion ratio within the membrane was calculated using the mass balance in the solution (eq S19). In Figures 3a,b, the ratio on the *y*-axis represents the concentration of the varying cation divided by that of the fixed cation: Na^+^/NH_4_^+^ ratio in the Na^+^-presoaked scenario and the NH_4_^+^/Na^+^ ratio in the NH_4_^+^-presoaked case. The modified Donnan dialysis model incorporates adsorbed ions within the membrane, establishing equilibrium based on a revised total concentration (eq 11).

**Figure 3:**
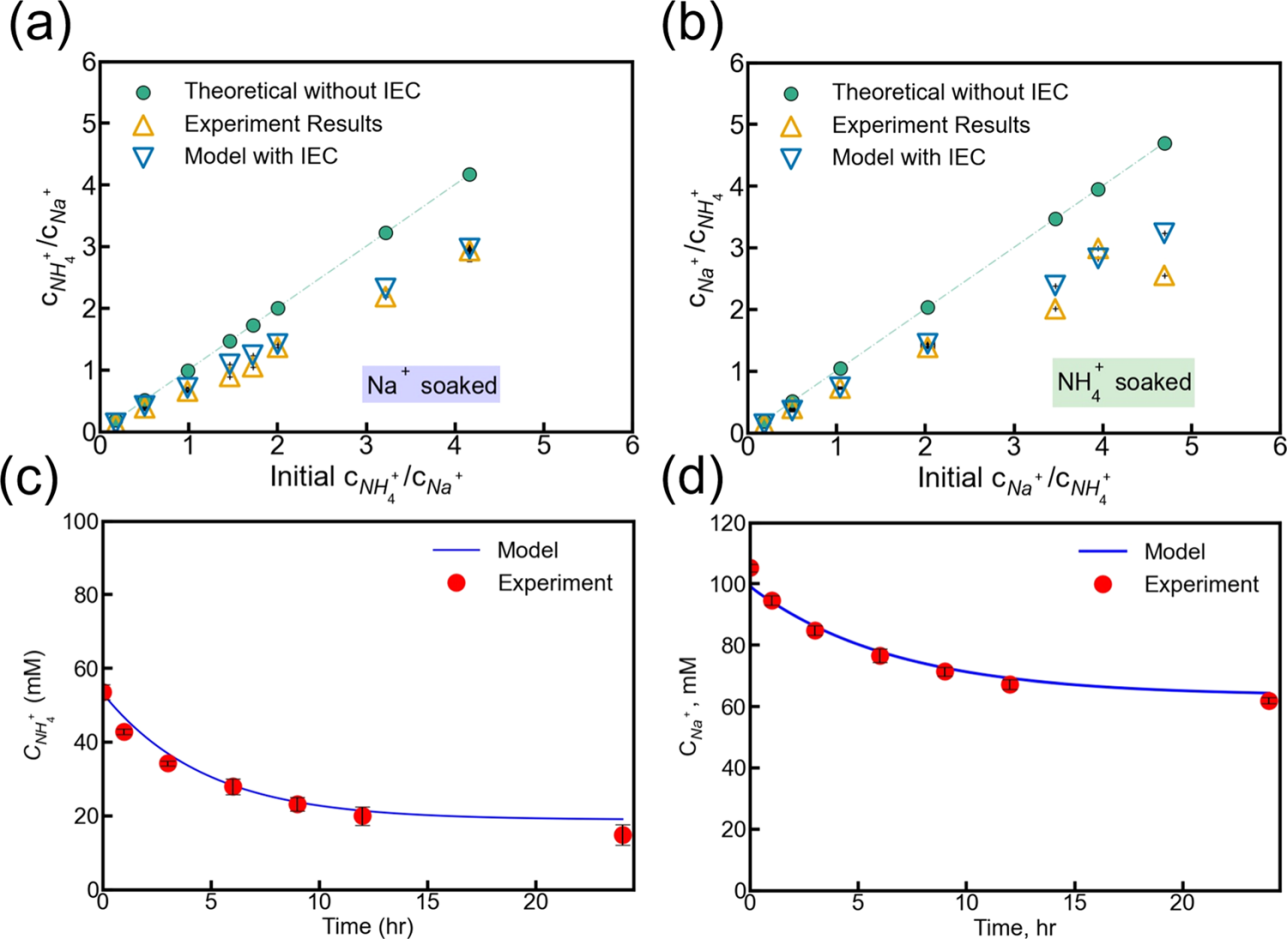
(a) The final NH_4_^+^/Na^+^ ratio in solution as a function of the initial concentration ratio based on theoretical calculations without IEC (green dots), experimental results (orange triangles), and the model with IEC (blue triangles) for Na^+^-presoaked membrane. (b) The final Na^+^/NH_4_^+^ ratio in solution as a function of initial concentration ratio based on theoretical calculations without IEC (green dots), experimental results (orange triangles), and the model with IEC (blue triangles) for NH_4_^+^-presoaked membrane. For (a,b), the dashed lines represent the 1:1 ratio, corresponding to the results obtained using DD without membrane capacity. (c) Concentration profile of NH_4_^+^ as a function of time for Na^+^-presoaked membrane. (d) Concentration profile of Na^+^ as a function of time for NH_4_^+^-presoaked membrane. For (c,d), red dots represent experimental data, while the solid line indicates model predictions.

Figure 3a,b shows comparisons among the theoretical ratios (without considering membrane uptake), experimental results, and predictions from the modified model. In the Na^+^-presoaked scenario, the original prediction (without incorporating ion exchange capacity, IEC) for the Na^+^/NH_4_^+^ ratio was 4.17, whereas the experimental result was 2.93, much lower than the original prediction. Our modified prediction is 2.96, which aligns closely with the experimental data. A similar improvement was observed for the NH_4_^+^/Na^+^ ratio with the NH_4_^+^-presoaked membrane: the experimental result (2.55) and modified prediction (3.23) were much closer compared to the initial prediction (4.69), demonstrating the effectiveness of our correction.

In both scenarios, the final concentration ratios in the solution deviate from the initial ones. This deviation becomes more significant as the concentration ratio of the nonpresoaked ion to the presoaked ion increases (Figure 3a,b, blue triangles). Mathematically, this occurs because membrane capacity is included in the denominator, amplifying deviations at higher concentrations of nonpresoaked cations. Additionally, higher solution concentrations reduce the water volume fraction in the membrane, enhancing membrane selectivity for NH_4_^+^ over Na^+^, likely due to the smaller hydrated ionic radius of NH_4_^+^ compared to Na^+^.^40^ Therefore, experimental data clearly align more closely with our modified DD model, highlighting the importance of accounting for the preoccupied cation content in the membrane. Furthermore, this approach provides a straightforward method to avoid complex estimations and minimize inaccuracies of high-capacity membranes in the Donnan dialysis process. For applicants, the predicted NH_4_^+^ concentration in the draw chamber when the membrane was presoaked by 1 M NH_4_Cl for different commercial CEMs show noticeable difference (Figure S4). As discussed in previous sections, the NH_4_^+^ ions presoaked in the membrane serve as an internal reservoir, facilitating additional NH_4_^+^ release into the draw solutions as the equilibrium shifts toward the NH_4_^+^ side. A higher IEC further enhances this effect by providing more exchange sites for NH_4_^+^ sorption and release, thereby increasing both the NH_4_^+^/Na^+^ ratio in the solution and the overall NH_4_^+^ transfer across the membrane. The comparison highlights the influence of different IEC values during the Donnan dialysis process. One limitation is that water uptake was only considered for the presoaking electrolytes and was treated as constant during the DD experiments, which may affect the quantitative data but is not expected to change the observed trends with membrane presoaking.

Figure 3c,d depicts the concentration profile of NH_4_^+^ in the feed solution and Na^+^ in the draw solution with a Na^+^-presoaked membrane and an NH_4_^+^-presoaked membrane, respectively. The nonsteady state approach successfully predicted the solution concentrations by accounting for ion accumulation in the membrane into account, which yields improved agreement with experimental data compared with conventional DD.^30^ For the Na^+^-presoaked membrane, the experimental concentration of NH_4_^+^ at the sixth hour was 29.26 mmol L^–1^, while the model predicted a concentration of 28.05 mmol L^–1^. Similarly, with the NH_4_^+^-presoaked membrane, the Na^+^ concentration in the draw solution at the sixth hour was 74.92 mmol L^–1^ experimentally, compared to a predicted value of 77.13 mmol L^–1^. The root-mean-square errors (RMSE) were 1.89 mmol L^–1^ and 3.00 mmol L^–1^ for the Na^+^- and NH_4_^+^-presoaked membranes, respectively (Text S2). These close agreements between model prediction and experimental data highlight the critical role of membrane sorption in accurately capturing the ion transport behavior.

### 4.5 Concentration Ratio in Membrane and in Solution

Figure 4a,b illustrates the changes in the concentration ratio of cations in the membrane to that in the solution before and after the experiment. The dashed line represents the ideal one-to-one ratio. The membrane concentrations were back calculated from the mass balance in the solution according to eq S19. In both Na^+^- and NH_4_^+^-presoaked conditions, the final membrane-to-solution concentration ratios in the solution deviate from the initial ones, with Na^+^-presoaked membranes exhibiting relatively smaller deviations. In Figure 4a, the maximum deviation occurs when the NH_4_^+^/Na^+^ ratio in the membrane reaches 1.16, whereas the corresponding value in the solution is 1.05. In contrast, under NH_4_^+^-presoaked conditions (Figure 4b), the final Na^+^/NH_4_^+^ ratio in the membrane is 2.27, while the solution ratio is 2.99.

**Figure 4:**
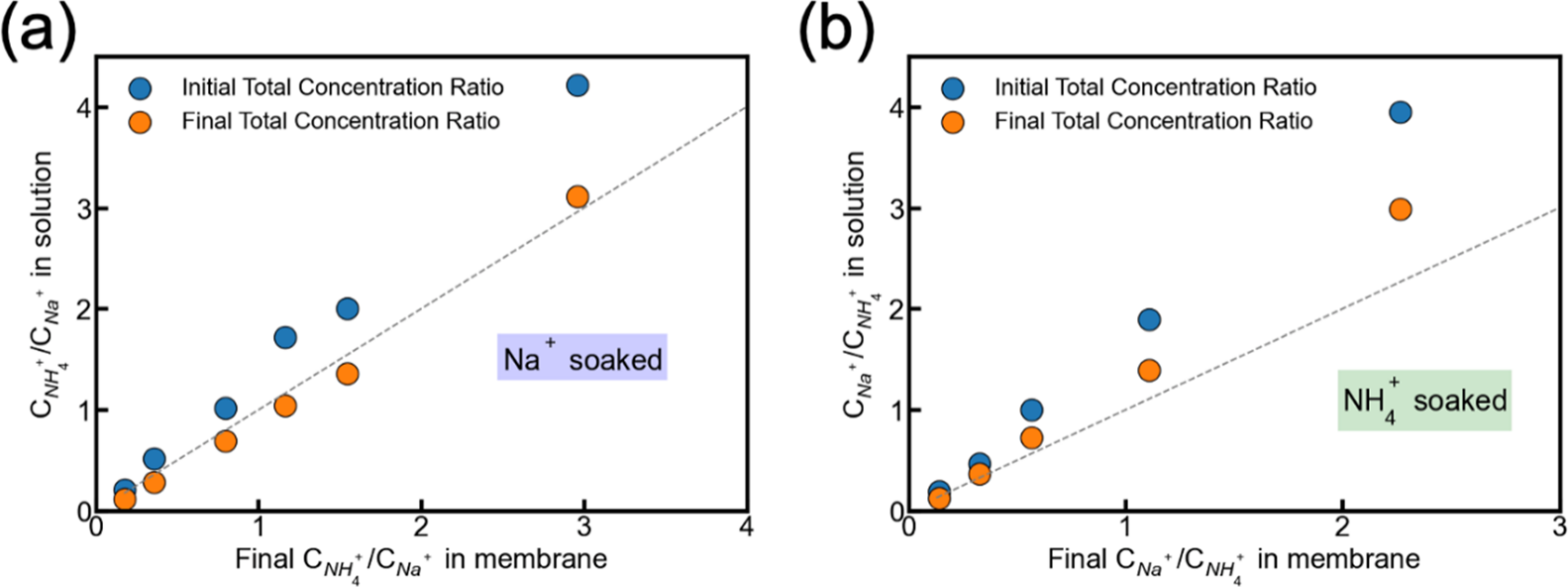
(a) The NH_4_^+^/Na^+^ ratio in solution as a function of the final concentration ratio in the membrane for Na^+^-presoaked membrane. The blue dots represent the initial solution concentration ratios, and orange dots represent final solution concentration ratios. (b) The Na^+^/NH_4_^+^ ratio in solution as a function of the final concentration ratio in the membrane for NH_4_^+^-presoaked membrane. The blue dots represent the initial solution concentration ratios, and the orange dots represent final solution concentration ratios. The dashed line represents the 1 to 1 ratio.

These discrepancies can be attributed to two main factors: differences in concentration ratios and differences in ion activity coefficients between the membrane and the solution. First, the ion sorption coefficient, defined as the ratio of a cation’s concentration in the membrane to that in the solution, varies between different cation species and affects the equilibrium between the solution and the membrane.^41^ Although only monovalent counterions and co-ions were used in the experiment, the different affinities of Na^+^ and NH_4_^+^ to the fixed charge group of the membrane caused the different sorption behaviors. Our ion sorption experiments indicated that at equilibrium conditions with 100 mmol L^–1^ NaCl as the feed and 50 mmol L^–1^ NH_4_Cl as the draw solution, the ion sorption selectivity of NH_4_^+^ over Na^+^ was 1.17 when the membrane was presoaked with NaCl. Conversely, the selectivity of Na^+^ over NH_4_^+^ was 0.76 when the membrane was presoaked with NH_4_Cl. This result aligns with the known cation selectivity order for sulfonic group CEMs (NH_4_^+^ > Na^+^), which is also reflected in Figure 4b, where the slope is greater than 1.^42^ The nature of the presoaked ion affects this selectivity, likely due to the tetrahedral geometry of the NH_4_^+^ ions, which enables stronger ionic interactions with sulfonic groups in the membrane. When the membrane is presoaked with NH_4_^+^, the fixed sites become more favorable for additional ammonium uptake, whereas when Na^+^ ions are presoaked, ammonium ions experience steric or electrostatic hindrance at the membrane–solution interface, reducing their sorption affinity and therefore lowering selectivity. The selectivity coefficient as a function of the initial total concentration was also calculated (Figure S5). A relatively larger variation in the selectivity coefficient was observed at lower total concentrations, whereas it remained nearly constant as the total concentration increased. Overall, the selectivity coefficient exhibits only minor variation across the investigated concentration range, indicating that concentration-dependent selectivity effects are limited under the experimental conditions of this study.

Second, the discrepancies are also influenced by differences in the activity coefficients of the cations in the membrane and in the solution. This effect has been previously demonstrated in Donnan dialysis processes involving single ionic species.^43,44,46^ Due to the nonideal behavior of ions within the membrane polymers, the ion activity coefficients are not always equal to unity.^45^ In particular, when the external solution concentration is low, the activity coefficients of ions inside the membrane are reduced.^44^ These variations in ion activity coefficients affect the partitioning and distribution of ions within the membrane and subsequently influence their equilibrium concentrations in the solutions. By taking the activity coefficient ratio in the model into account (eq S5), we concluded that the ratio between the activity coefficients of the cation and the presoaked cation in the solution is consistently larger than that in the membrane. Moreover, NH_4_^+^ is more prone to forming ion pairs, which facilitate its transport through the membrane, in contrast to NaCl, which dissociates completely in solution and lacks such ion-pairing behavior.^46^ Unlike fully dissociated co-ions, ion paired species are less subject to electrostatic exclusion, which makes it easier to cross the membrane.^3^ Overall, these findings suggest that the observed deviation between membrane and solution ion ratios is governed not only by concentration differences but also by ion-specific interactions, possibly including activity coefficients, sorption affinities, and the nature of the presoaked ions.

## 5 Conclusions

In this study, we identified membrane ion sorption as a key factor contributing to the discrepancy between theoretical predictions and experimental observations of the Donnan dialysis (DD) process. In the experiments, the NH_4_^+^ fraction with a presoaked cation exchange membrane derived from the theoretical value, indicating the ion exchange capacity in the DD process cannot be neglected. Therefore, we proposed a modified DD equation that incorporates the membrane capacity, enabling a more accurate representation of the ion exchange process.

By tracking the concentration changes over time in both the solution and the membrane, we confirmed that the total ion concentration in solution shifted away from the theoretical values calculated by mass balance in the solution due to sorption and desorption behavior. The modified model demonstrated improved agreement with the experimental data compared with the conventional approach. Furthermore, a nonsteady state kinetics model was further employed to quantify the interdiffusion behavior across the membrane. In addition, the deviation in the Na^+^/NH_4_^+^ ratio between the membrane and the solution can be attributed to differences in ion affinity to the membrane fixed charge group, variations in activity coefficients, and possibly the influence of the ion-pairing effect. It should be also noted that the specific commercial CEM was chosen; further application to other specify membrane (e.g., higher permeability membranes and typical selective membranes) will be explored in future work.

Overall, our study demonstrates how ion sorption alters Donnan equilibrium during dialysis and emphasizes that equilibrium is ultimately reached based on the new total ion concentrations established by membrane–solution interactions. Future studies should focus on comparing ammonium with other ions, not only due to its agricultural value as a fertilizer but also due to its unique tetrahedral geometry, which differentiates it from spherical cations, and its unique characteristics when transporting in a cation exchange membrane.

## Supplementary Material



## Notes

Detailed formulation of the modified Donnan equation, input parameters used in the model, root-mean-square error calculation method, cation concentrations in the draw and feed under different membrane presoaked conditions and draw solution concentrations, molar differences between initial and final states, model based predictions of the final NH_4_^+^ concentration in the draw chamber of DD using different commercial CEMs, manufacturers’ specifications of commercial CEMs, selectivity coefficient as a function of the initial total concentration, and ion amounts within the membrane profile under different presoaked cation conditions (PDF)
