# Supplementary material for: Influence of Membrane Ion Sorption on Ammonium Transport in Donnan Dialysis with Cation Exchange Membranes
Source: ACS ES T Eng. 2026 Jan 30;6(2):909–19. doi: 10.1021/acsestengg.5c01024 (PMC12910581; doi:10.1021/acsestengg.5c01024)
Supplement: Supplementary file 1 [file ee5c01024_si_001.pdf]

Supporting Information

Influence of Membrane Ion Sorption on Ammonium Transport in Donnan Dialysis with Cation Exchange Membranes

Hanyu Tang, Kai Yang, Mohan Qin*

Department of Civil and Environmental Engineering, University of Wisconsin–Madison, Madison, Wisconsin 53706, USA

* Corresponding author. E-mail: mohan.qin@wisc.edu

Text S1: Modified Donnan Equation calculation for ion concentration at equilibrium

At Donna equilibrium, the electrochemical potential ($\bar{\mu}$) of a typical charged species, i, in the feed (*f*) and draw (*p*) side at time *t*, is equal:

$$\overline{\mu_i^f} = \overline{\mu_i^p} \qquad \text{Eq. S1}$$

This can be expressed as:

$$\mu_i^0 + RTln a_{i,p,t} + z_i F\psi_{p,t} = \mu_i^0 + RTln a_{i,f,t} + z_i F\psi_{f,t} \qquad \text{Eq. S2}$$

where μ is chemical potential, R is the gas constant, T is the absolute temperature, a is ionic activity, z is the ion valence, F is Faraday's constant, ψ is the electrical potential, 0 in superscript means standard state. The equation can be simplified into the following:

$$\frac{z_i F(\psi_{p,t} - \psi_{f,t})}{RT} = ln\left(\frac{a_{i,f,t}}{a_{i,p,t}}\right) \qquad \text{Eq. S3}$$

In this process, i can be Na^+ or NH_4^+, and Eq 2 can be converted into the following:

$$\frac{z_{Na^+} F(\psi_{p,t} - \psi_{f,t})}{RT} = ln\left(\frac{a_{Na^+,f,t}}{a_{Na^+,p,t}}\right) \qquad \text{Eq. S4}$$

$$\frac{z_{NH_4^+} F(\psi_{p,t} - \psi_{f,t})}{RT} = ln\left(\frac{a_{NH_4^+,f,t}}{a_{NH_4^+,p,t}}\right) \qquad \text{Eq. S5}$$

For a known scenario, $\psi_{p,t} - \psi_{f,t}$ is the same when the solution between the membrane is ensured. As a result, the left sides of Eq S4 and S5 are equivalent and can be further expressed as follows:

$$\frac{a_{Na^+,f,t}}{a_{Na^+,p,t}} = \frac{a_{NH_4^+,f,t}}{a_{NH_4^+,p,t}} \quad \text{Eq. S6}$$

The equation can be further converted as the following because of $a = \gamma \cdot C$:

$$\frac{\gamma_{Na^+,f,t} \cdot C_{Na^+_{f,t}}}{\gamma_{Na^+,p,t} \cdot C_{Na^+_{p,t}}} = \frac{\gamma_{NH_4^+,f,t} \cdot C_{NH_4^+{}_{f,t}}}{\gamma_{NH_4^+,p,t} \cdot C_{NH_4^+{}_{p,t}}} \quad \text{Eq. S7}$$

We assumed the activity coefficient ratios between the feed and draw solutions for both Na^+ and NH_4^+ are similar under conditions studied, so the equation can be further simplified as the following:

$$\frac{C_{Na^+_{f,t}}}{C_{Na^+_{p,t}}} = \frac{C_{NH_4^+{}_{f,t}}}{C_{NH_4^+{}_{p,t}}} \quad \text{Eq. S8}$$

Accordingly, the equation is still found when two sides are multiplied by the ratio of the volume:

$$\frac{C_{Na^+_{f,t}}}{C_{Na^+_{p,t}}} \cdot \frac{V_f}{V_p} = \frac{C_{NH_4^+{}_{f,t}}}{C_{NH_4^+{}_{p,t}}} \cdot \frac{V_f}{V_p} \quad \text{Eq. S9}$$

Because the solution volume ratio of feed side to draw side is 1:1, the equation can be written as:

$$\frac{n_{Na^+_{f,t}}}{n_{Na^+_{p,t}}} = \frac{n_{NH_4^+{}_{f,t}}}{n_{NH_4^+{}_{p,t}}} \quad \text{Eq. S10}$$

Similarly, the ratio of ion concentration in membrane and that in the solution can be deducted using the same approach. Here, we assume that different ions have the same activity coefficient within the membrane. The equation can be described as follows:

$$\frac{c_i^m}{c_i^p} = \frac{c_j^m}{c_j^p} \qquad \text{Eq. S11}$$

where i, j represents different ions in the system.

To further predict the final stage concentration by using the initial status, mass balance in the solution is used in the following equations:

$$n_{NH_4^+{}_{f,t}} + n_{NH_4^+{}_{p,t}} = n_{NH_4^+{}_{total,t}} \qquad \text{Eq. S12}$$

$$n_{Na^+_{f,t}} + n_{Na^+_{p,t}} = n_{Na^+_{total,t}} \qquad \text{Eq. S13}$$

where $n_{i_{total,t}}$ is the mass of total species i in the solution at Donnan equilibrium. The mass of the typical species in the solution can be further described as the initial mass in the solution and the difference from membrane uptake or release

$$n_{NH_4^+{}_{total,t}} = n_{NH_4^+{}_{f,0}} + \Delta n_{NH_4^+{}_t} \qquad \text{Eq. S14}$$

$$n_{Na^+{}_{total,t}} = n_{Na^+{}_{p,0}} + \Delta n_{Na^+{}_t} \qquad \text{Eq. S15}$$

Here, Δn_{i_t} denotes the net change of specie i due to ion exchange with the membrane up to Donnan equilibrium. A positive Δn_{i_t} indicates a net release from the membrane to the solution, whereas a negative value indicates a net uptake by the membrane:

$$\Delta n_{Na^+_t} = IEC - n_{Na^+_{mi,t}} \qquad \text{Eq. S16}$$

$$\Delta n_{NH_4^+{}_t} = -n_{NH_4^+{}_{mi,t}} \qquad \text{Eq. S17}$$

where IEC is the ion exchange capacity for a known area membrane. Because the ratio between Na^+ and NH_4^+ is the same inside the membrane and in the solution, the equation can be further converted into the following:

$$\frac{n_{NH_4^+ mi,t}}{n_{Na^+_{mi,t}}} = \frac{n_{NH_4^+ total,t}}{n_{Na^+_{total,t}}} = \frac{n_{NH_4^+ f,0} + \Delta n_{NH_4^+ m,t}}{n_{Na^+_{p,0}} + \Delta n_{Na^+_{m,t}}}$$

$$= \frac{n_{NH_4^+ f,0} + \Delta n_{NH_4^+ m,t} + n_{NH_4^+ mi,t}}{n_{Na^+_{p,0}} + \Delta n_{Na^+_{m,t}} + n_{Na^+_{mi,t}}} \qquad \text{Eq. S18}$$

The equation can be further modified according to Eq. S14 and S15, the final ratio in the solution can be predicted

$$\frac{n_{NH_4^+ total,t}}{n_{Na^+_{total,t}}} = \frac{n_{NH_4^+ f,0}}{n_{Na^+_{p,0}} + IEC} \qquad \text{Eq. S19}$$

When the membrane was presoaked by NH_4Cl, it can be written as

$$\frac{n_{NH_4^+ total,t}}{n_{Na^+_{total,t}}} = \frac{n_{NH_4^+ f,0} + IEC}{n_{Na^+_{p,0}}} \qquad \text{Eq. S20}$$

The concentration of species i in the solution can be written as the sum of its concentrations in the draw and feed solutions, due to the equal volumes in both chambers:

$$C_{solution,\ i} = C_{draw,i} + C_{feed,i} \qquad \text{Eq. S21}$$

The mass balance of the spices that were initially absent from the membrane can be described as the total amount in the system, which is the sum of their amounts in the liquid phase and in the membrane at equilibrium:

$$n_{solution,0} = n_{liquid,t} + n_{membrane,t} \quad \text{Eq. S22}$$

The input parameters used in kinetics modeling are listed in Table S1.

Table S1. Input parameters used in the model

Input parameters	
Ion exchange capacity, mmol	5.12
Membrane thickness, cm	0.049
D_{Na^+}, $cm^2\ s^{-1}$	1.33×10^{-6}
$D_{NH_4^+}$, $cm^2\ s^{-1}$	1.96×10^{-6}
$K_{NH_4^+}^{Na^+}$	0.76
$K_{Na^+}^{NH_4^+}$	1.17

Text S2. Root Mean Square Error (RMSE)

RMSE is used to evaluate the difference between the experimental number and the model prediction, which can be expressed as follow:

$$RMSE = \sqrt{\frac{1}{n}\sum_{i=1}^{n}(S_i - O_i)^2}$$

where S_i is the model prediction, and O_i is the experimental data.

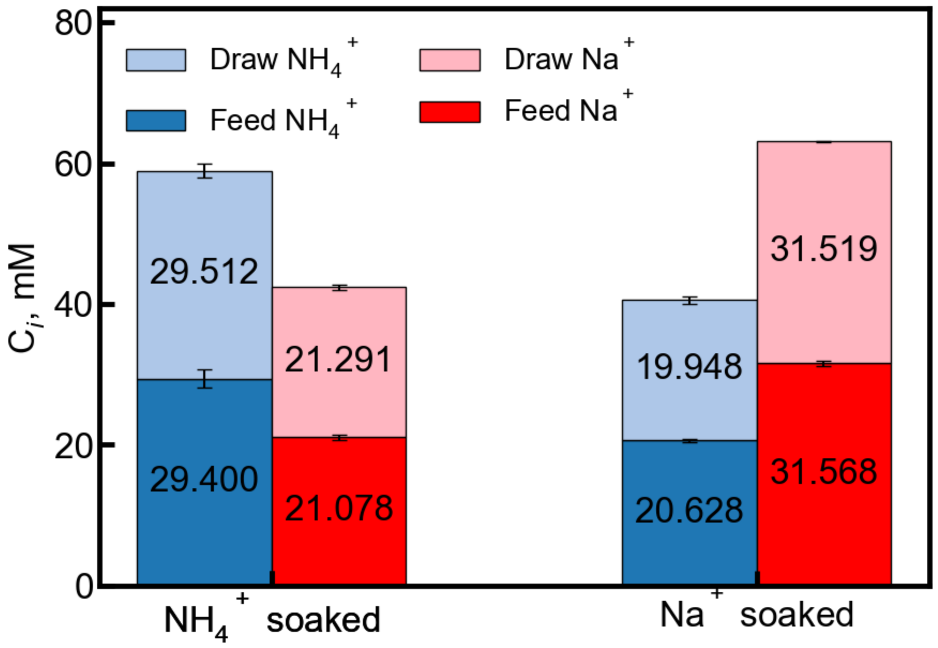

Figure S1. The concentrations of NH_4^+ and Na^+ after 24 hours in each chamber when the initial feed solution was 50 mmol L^{-1} NH_4Cl and the draw solution was 50 mmol L^{-1} NaCl.

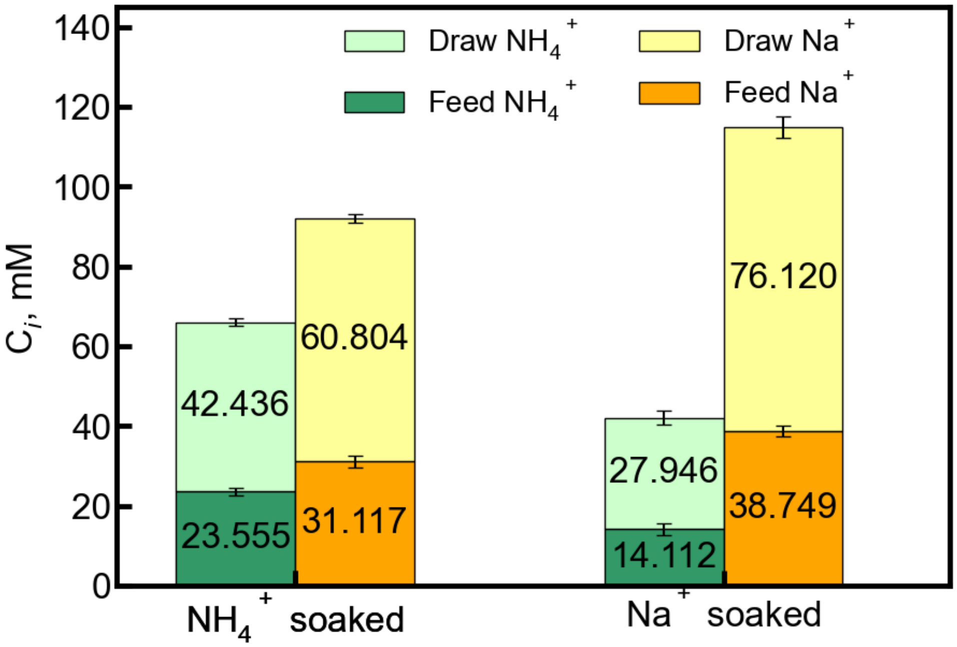

Figure S2. The concentrations of NH_4^+ and Na^+ after 24 hours in each chamber when the initial feed solution was 50 mmol L^{-1} NH_4Cl and the draw solution was 100 mmol L^{-1} NaCl.

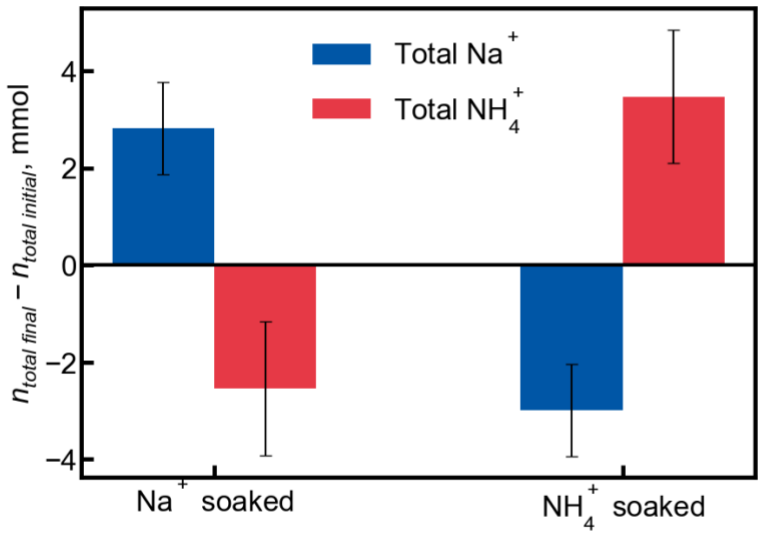

Figure S3. Molar difference between the initial and the final states in the system of Na^+ and NH_4^+, with membranes presoaked in Na^+ and NH_4^+ solutions.

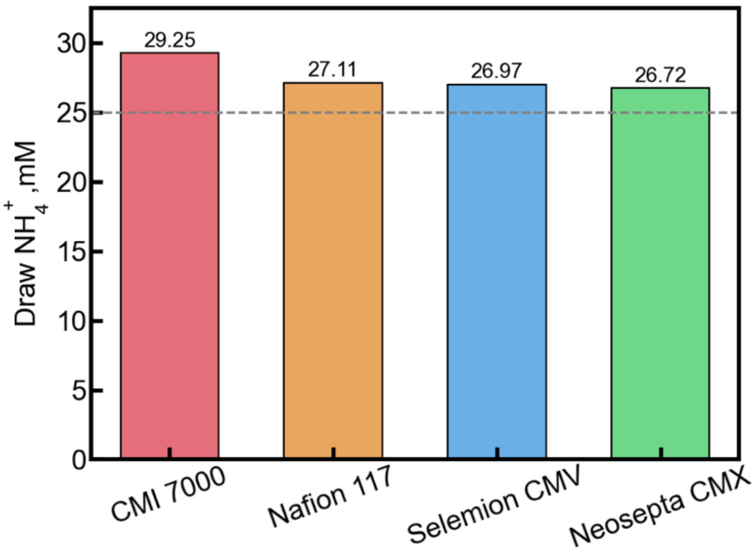

Figure S4. Final NH_4^+ concentration in the draw chamber of DD with different commercial CEMs. All the CEMs were presoaked by 1M NH_4Cl overnight. The feed and draw solutions are 50 mM NH_4Cl and 50 mM NaCl, respectively. The dashed line indicates the value predicted by original Donnan Dialysis model (25 mM). The IEC and other properties of the membranes (Nafion 117, Selemion CMV, and Neosepta CMX) were obtained from the manufacturers' specifications and listed in Table S2.

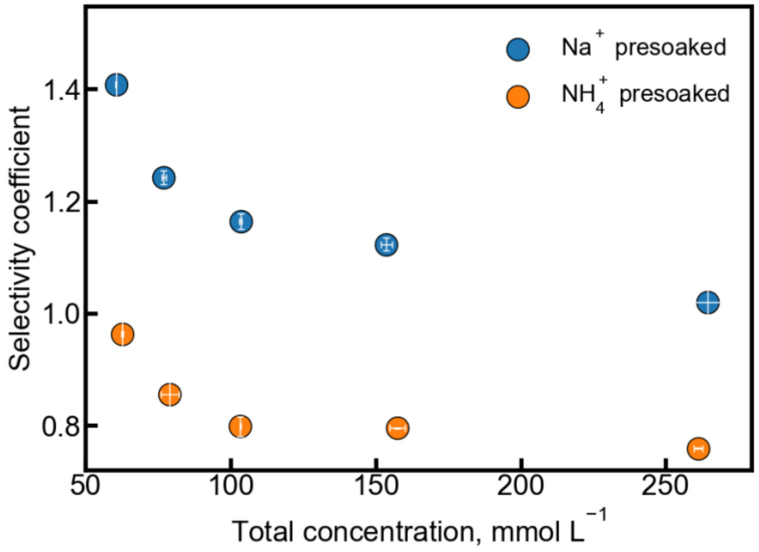

Figure S5. Selectivity coefficient as a function of the initial total concentration (feed + draw) in the external solution (feed + draw). The selectivity coefficient is defined as $\frac{C_{m,A}/C_{m,B}}{C_{s,A}/C_{s,B}}$, where B is the presoaked ion, and A is the other ion.

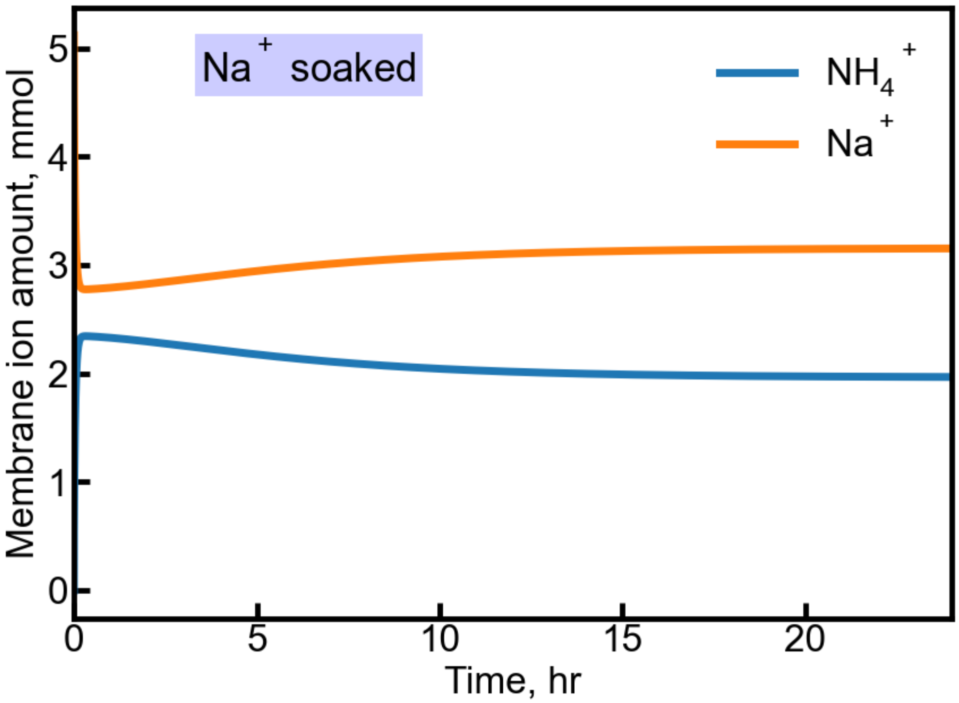

Figure S6. Ion amounts within the membrane as a function of time for the membrane presoaked by 1 mol L^{-1} NaCl, as predicted by the non-steady-state model.

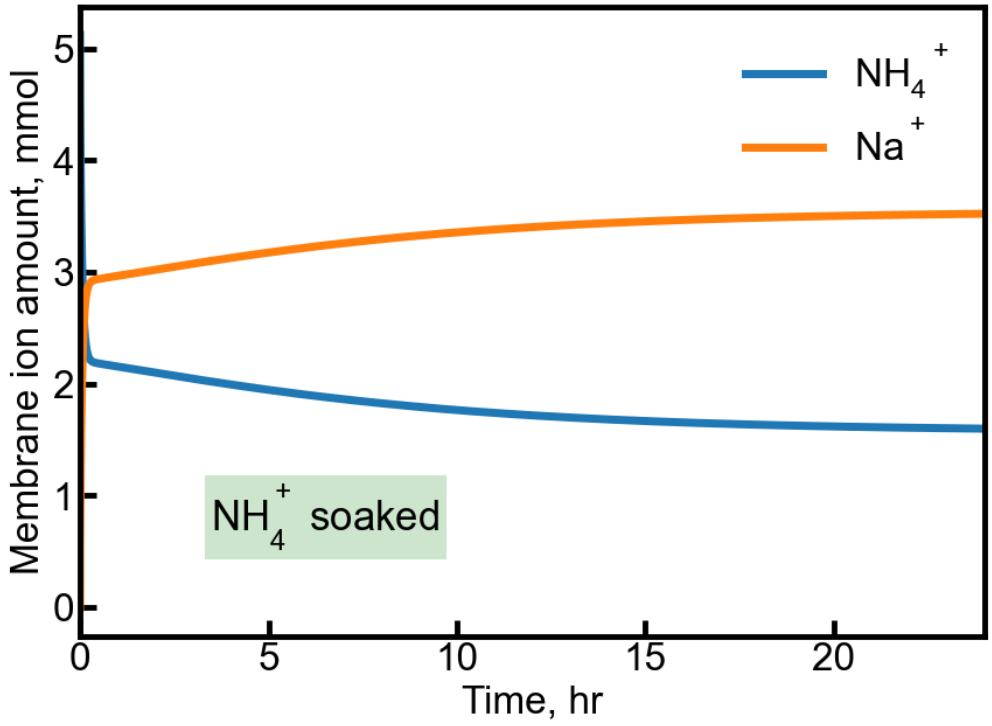

Figure S7. Ion amounts within the membrane as a function of time for the membrane presoaked by 1 mol L^{-1} NH_4Cl, as predicted by the non-steady-state model.

Table S2. Manufacturers' specification of the commercial CEMs

	Ion Exchange Capacity, meq g^{-1} dry membrane	Dry density, g cm^{-3}	Thickness, μm
Nafion 117	0.91	1.98	183[1]
Selemion CMV	2.4	1.2	116
Neosepta CMX	1.65	1[a]	175

[a] Not explicitly reported; inferred from a comparable membrane.

References

1. P. Szczepański, G. Szczepańska, Donnan dialysis − A new predictive model for non−steady state transport, J. Membr. Sci. 525 (2017) 277–289. https://doi.org/10.1016/j.memsci.2016.11.017.
